# Efficient Uptake of Recombinant Lipidated Survivin by Antigen-Presenting Cells Initiates Antigen Cross-Presentation and Antitumor Immunity

**DOI:** 10.3389/fimmu.2018.00822

**Published:** 2018-04-23

**Authors:** Chen-Yi Chiang, Yi-Jyun Chen, Chiao-Chieh Wu, Shih-Jen Liu, Chih-Hsiang Leng, Hsin-Wei Chen

**Affiliations:** ^1^National Institute of Infectious Diseases and Vaccinology, National Health Research Institutes, Miaoli, Taiwan; ^2^Graduate Institute of Biomedical Sciences, China Medical University, Taichung, Taiwan

**Keywords:** antigen-presenting cells, cancer immunotherapy, CD8 T cells, cross-presentation, recombinant lipoprotein, survivin

## Abstract

Survivin is overexpressed in various types of human cancer, but rarely expressed in terminally differentiated adult tissues. Thus, survivin is a potential target antigen for a cancer vaccine. However, self-tumor-associated antigens are not highly immunogenic. Bacteria-derived lipoproteins can activate antigen-presenting cells through their toll-like receptors to enhance immune responses. In this context, lipidated survivin is an attractive candidate for cancer immunotherapy. In the present study, recombinant lipidated human survivin (LSur) was prepared from an *Escherichia coli*-based system. We investigated whether LSur is efficiently captured by antigen-presenting cells then facilitating effective induction of survivin cross-presentation and generation of immunity against cancer cells. Our results demonstrate that LSur, but not its non-lipidated counterpart, can activate mouse bone-marrow-derived-dendritic cells (BMDCs) to enhance cytokine (IL-6, TNF-α, and IL-12) secretion and costimulatory molecules (CD40, CD80, CD86, and MHC II) expression. However, the pathways involved in the capture of the recombinant lipidated antigen by antigen-presenting cells have not yet been elucidated. To this end, we employ various endocytosis inhibitors to study the effect on LSur internalization. We show that the internalization of LSur is suppressed by the inhibition of various routes of endocytosis. These results suggest that endocytosis of LSur by BMDCs can be mediated by multiple mechanisms. Furthermore, LSur is trafficked to the early endosome after internalization by BMDCs. These features of LSur are advantageous for cross-presentation and the induction of antitumor immunity. We demonstrate that immunization of C57BL/6 mice with LSur under treatment with exogenous adjuvant-free formulation induce survivin-specific CD8^+^ T-cell responses and suppress tumor growth. The antitumor responses are mediated by CD8^+^ cells. Our findings indicate that LSur is a potential candidate for stimulating protective antitumor immunity. This study suggests that lipidated tumor antigens may be a promising approach for raising a robust antitumor response in cancer immunotherapy.

## Introduction

Survivin is a member of the inhibitor of apoptosis (IAP) family of proteins, which prevent apoptosis and promote cell proliferation (1–3). It has been shown that the expression of survivin (Sur) is highly tumor-specific, and Sur is rarely expressed in terminally differentiated adult tissues (4, 5). Sur is overexpressed in various types of human cancer, such as lung (6, 7), breast (8, 9), prostate (10), and ovarian (11, 12) cancers. Furthermore, Sur is upregulated not only in cancer cells but also in the tumor-associated stroma (13). Sur is a growth factor-inducible gene that is strongly overexpressed in actively dividing endothelial cells during blood vessel formation, a process in which Sur plays an important role in offsetting apoptotic stimuli and stabilizing the vascular network (14–17). Based on these features, Sur may be a universal target antigen for a cancer vaccine. The downregulation of Sur would severely inhibit the survival capacity of tumor cells and would be beneficial to patients. Several reports have shown that high tumor levels of Sur are associated with adverse outcomes in patients with different types of cancer (4–6, 8–12). These results suggest that Sur is an ideal target candidate for anticancer immunotherapy because it is not subject to immune selection (the selection of cancer cells not expressing Sur).

The rationale of a therapeutic cancer vaccine is to target a specific tumor antigen then trigger the patient’s own immune defense system to eliminate cancer cells. However, the induction of a robust immune response to self-tumor-associated antigens has thus far been difficult to achieve (18, 19). Dendritic cells (DCs) are recognized as the most potent antigen-presenting cells that initiate antigen-specific immune responses. Several pattern recognition receptors, such as toll-like receptors, are expressed by DCs to sense pathogen-associated molecular patterns in the cellular environment. DCs patrol peripheral tissues and capture antigens that can then be processed and presented by both class-I and -II MHCs to stimulate antigen-specific T cells (20–23). Therefore, the expression of self-tumor-associated antigens with pathogen-associated molecular patterns will increase the immunogenicity of self-tumor-associated antigens.

Bacterial lipoproteins have been shown to activate antigen-presenting cells through their toll-like receptors (24–29). The activation of antigen-presenting cells can upregulate costimulatory molecules and cytokine production, which can enhance immune responses. However, recombinant lipoproteins have not been fully utilized as vaccine candidates due to low lipoprotein expression levels. Incomplete modification and the absence of lipidation are two common problems encountered when lipoproteins are overexpressed in *Escherichia coli* (30–32). Thus, obtaining high yields of lipoproteins for large-scale production remains a great challenge. In our previous studies, we established a novel platform for expressing high levels of recombinant lipoproteins using an *E. coli*-based system (33). Using this technology, we can both prepare lipidated vaccine candidates and convert non-lipoproteins into lipoproteins. These recombinant lipidated vaccine candidates have been shown to induce robust immune responses without exogenous adjuvant formulation (34–36).

In this study, human Sur was used as a model antigen and were expressed in the lipidated form. We hypothesized that recombinant lipidated human survivin (LSur) is a cancer vaccine candidate with built-in adjuvant activity which is able to enhance Sur-specific immune responses. The merit of LSur was validated by showing the accessibility to DCs, intracellular trafficking pathways, and induction of antitumor immunity.

## Materials and Methods

### Mice and Cell Lines

Female C57BL/6 mice were obtained from the National Laboratory Animal Center, Taipei, Taiwan. All of the mice were housed at the Laboratory Animal Center of the National Health Research Institutes. All animal studies were approved and were performed in compliance with the guidelines of the Animal Committee of the National Health Research Institutes.

The TC-1 cell line, a primary lung epithelial cell line from C57BL/6 mice immortalized with HPV-16 E6/E7 and the oncogene c-Ha-ras, was a kind gift from Dr. T-C. Wu (Johns Hopkins University, USA) (37). The cells were cultured in RPMI-1640 medium supplemented with 10% (v/v) heat-inactivated fetal bovine serum, penicillin/streptomycin (50 units/mL), sodium pyruvate (0.5 mM), HEPES (20 mM), and β-mercaptoethanol (50 µM) at 37°C under 5% CO_2_.

### Antibodies and Reagents

The FITC-, PE-conjugated antibodies specific to CD8, CD11c, CD40, CD80, CD86, and MHC II were purchased from BD Biosciences (San Diego, CA, USA). The purified anti-CD8 and isotype control antibodies were purchased from eBioscience (San Diego, CA, USA). The biotinylated goat antihuman Sur antibody was purchased from R&D (R&D System Inc., MN, USA). The alexa 488-conjugated antibodies against EEA1 (Abcam, UK) were used to perform compartment staining. Hoechst 33342, alexa 488-conjuated phalloidin and alexa 594-conjugated streptavidin were purchased from Molecular Probes (Thermo Fisher Scientific, MA, USA). DAKO fluorescent mounting medium was purchased from Agilent Technologies (CA, USA).

### Construction of Expression Vectors

Based on the amino-acid sequence of human Sur, the DNA sequence of Sur (AAC51660) using *E. coli* codons was determined and fully synthesized by Mission Biotechnology Co. (Taipei, Taiwan). The forward primer (5′-GATATACATATGATGGGCGCGCCGACCCT-3′, *Nde*I site is underlined) and reverse primer (5′-GTGGTGCTCGAGATCCATCGCCGCCAG-3′, *Xho*I site is underlined) were used to amplify the synthetic DNA. The PCR product was then cloned into the *Nde*I and *Xho*I sites of the expression vector pET-22b(+) (Novagen, Madison, WI, USA) to produce the plasmid pSurvivin. To clone and express LSur, the D1 domain and the lipid signal peptide of the lipoprotein Ag473 (33) were cloned into the *Nde*I and *Bam*HI sites of the pET-22b(+) expression vector (Novagen, Madison, WI, USA) to obtain the plasmid pLipo. The forward primer (5′-GATATAGGATCCATGGGCGCGCCGACCCT-3′, *Bam*HI site is underlined) and the previous reverse primer were used to amplify the Sur gene. The PCR product was cloned into the *Bam*HI and *Xho*I sites of the pLipo plasmid to produce the plasmid pLSur. As a result, the C-terminus of recombinant human Sur and LSur contained a hexahistidine tag (His-tag).

### Production and Purification of Recombinant Proteins

To express Sur, *E. coli* BL21 (DE3) (Invitrogen, Carlsbad, CA, USA) was transformed with pSurvivin. The transformed cells were cultured at 37°C overnight, and protein expression was induced by adding 1-mM isopropylthiogalactoside (IPTG), followed by incubation for 3 h at 37°C. To express LSur, *E. coli* C43(DE3) (Lucigen, Middleton, WI, USA) was transformed with pLSur to express the lipidated protein. The transformed cells were cultured at 37°C overnight. One 15 mL of the overnight culture was scaled up to 600 mL in a 2-L-shake flask and incubated at 20°C for 4 h before induction. Protein expression was induced (OD_600_ = 0.8) by adding 1-mM IPTG, followed by incubation at 20°C for 20 h.

Survivin was purified by disrupting the harvested cells in a French press (Constant Systems, Daventry, UK) at 27 Kpsi in homogenization buffer [20-mM Tris (pH 8.0), 40-mM sucrose, 400-mM NaCl and 10% glycerol]. The cell lysate was clarified by centrifugation (80,000× *g* for 40 min). Most of the Sur was present in inclusion bodies. Sur was then solubilized with extraction buffer [20-mM Tris (pH 8.0), 40-mM sucrose, 400-mM NaCl, 10% glycerol, 20-mM imidazole, and 3-M guanidine hydrochloride]. The extracted fraction was loaded onto immobilized metal affinity chromatography (IMAC) columns (BIO-RAD, Hercules, CA, USA, 2.5-cm i.d. × 10.0 cm) containing 20-mL Ni-NTA resin (Qiagen, San Diego, CA, USA) to purify Sur. The column was washed twice with the extraction buffer. Then, Sur was eluted with the homogenization buffer containing 500-mM imidazole. The eluted Sur was dialyzed using 20-mM Tris (pH 8.0) three times for at least 6 h each time. After dialysis, an E membrane (Pall Co., NY, USA) was used to remove endotoxin. After dialysis against 50-mM ammonia bicarbonate (pH 8.0), the Sur was lyophilized and stored at −20°C. The disruption and purification steps in the production of LSur were similar to those used for Sur. LSur was extracted from the pellet using solubilization buffer [1% Triton X-100 and 20-mM Tris (pH 8.0)]. The extraction supernatant was collected by centrifugation. The supernatant was incubated with 25 mL of copper chelating sepharose (GE Healthcare, IL) and loaded onto a column. The column was washed with the washing buffer [1% Triton X-100, 0.4-M NaCl and 50-mM Tris (pH 8.9)] followed by the same buffer containing 20-mM imidazole, and then washed with a 100-fold column volume of 50-mM Tris (pH 8.9) and 0.4-M NaCl containing 0.1% Triton X-114 to remove the lipopolysaccharide (LPS). Next, the column was washed without 0.1% Triton X-114 to remove the residual detergent, and LSur was eluted with 50-mM Tris (pH 8.9) containing 500-mM imidazole. The solubilization buffer was exchanged with 50-mM Tris (pH 8.9). The fractions from each step were analyzed by SDS-PAGE and immunoblotted with anti-His-tag antibodies. The endotoxin levels of the purified Sur and LSur samples were determined using the Limulus amebocyte lysate (LAL) assay (Associates of Cape Cod, Inc., Cape Cod, MA, USA).

### Identification of the Lipid Moiety in LSur

Recombinant lipidated human survivin was digested with trypsin (Sigma-Aldrich, St. Louis, MO, USA). The reaction mixture was further purified with a ZipTip (Millipore, MA, USA) after digestion. A 1-µL aliquot of the ZipTip-polished tryptic fragments was mixed with 1 mL of a saturated solution of α-cyano-4-hydroxycinnamic acid in acetonitrile/0.1% trifluoroacetic acid (1:3 vol:vol). The mixture (1 µL) was placed on the target plate of an MALDI micro MX mass spectrometer (Waters, Manchester, UK) for analysis.

### Effect of LSur on Dendritic Cell Activation

The femurs and tibiae of C57BL/6 mice were removed and the bone marrow cells were dispersed by vigorous pipetting. After removing red blood cells with lysis buffer, the isolated bone marrow cells were resuspended (2–5 × 10^5^ cells/mL) with RPMI-1640 supplemented with 10% (v/v) heat-inactivated fetal bovine serum, penicillin/streptomycin (50 units/mL), L-glutamine (2 mM), HEPES (20 mM), and β-mercaptoethanol (50 µM) at 37°C under 5% CO_2_. On days 0 and 3, granulocyte macrophage colony stimulating factor (200 units/mL) was added to the cultures. Cultured cells were harvested on day 6. One-mL aliquots of suspended bone-marrow-derived dendritic cells (BMDCs) (1 × 10^6^ cells/mL) were seeded into 24-well plates and stimulated with Sur or LSur (2.5 or 10 µg/mL). The supernatants were collected at 20 h after stimulation. Cytokine (TNF-α, IL-6, IL-12p40, and IL-12p70) levels in the supernatants were determined using specific cytokine kits purchased from eBioscience (San Diego, CA, USA). Cells were harvested for surface marker staining using anti-CD11c, CD40, CD80, and CD86 MHC II monoclonal antibodies after 20 h of stimulation. The expression of surface markers was analyzed by flow cytometry (FACSCalibur, BD Biosciences) on gated CD11c^+^ cell populations. The data were acquired using the CellQuest Pro software and analyzed using FACS 3 software.

### Laser Scanning Confocal Microscopy

To study antigen internalization and localization, BMDCs were incubated with Sur or LSur (10 µg/mL) at 37°C for the indicated time. After incubation and washing to remove free antigens, the cells were fixed with 3.5% paraformaldehyde (Sigma-Aldrich) for 15 min at room temperature and then permeabilized in 0.1% saponin/PBS (eBioscience). After blocking with 3% BSA (Sigma-Aldrich) in PBS, the cells were stained with goat biotinylated antihuman Sur antibody (R&D System Inc., MN, USA) for 4 h. The samples were incubated with alexa 594-conjugated streptavidin at room temperature for 1 h. The cell compartments were then stained using alexa 488-conjugated phalloidin or anti-EEA1 antibodies at room temperature for 1 h. Nuclei were stained with Hoechst 33342 at room temperature for 15 min. The cells were air-dried and mounted with DAKO fluorescent mounting medium. After washing with wash buffer [1% FBS, 0.1% sodium azide and 2-mM EDTA in PBS], the cells were incubated with streptavidin-alexa 594 reagents (Thermo Fisher Scientific, MA, USA) at room temperature for 1 h. The cells were visualized using a Leica TCS SP5 II confocal microscope (Leica Microsystems, Heidelberg, Germany). Image acquisition and measurements of the colocalization rate were conducted using the Advanced Fluorescence software from Leica Microsystems. The colocalization rate was calculated using the following formulas: colocalization rate = area of colocalization/area of foreground, and foreground = area of image/area of background.

### Cytotoxicity of Endocytosis Inhibitors

To establish an optimal protocol for the use of endocytosis inhibitors on DCs, it was imperative to evaluate their *in vitro* cellular toxicity. We investigated the viability of DCs after exposure to four frequently used inhibitors: chlorpromazine, cytochalasin D, 5-(*N*-ethyl-*N*-isopropyl)-amiloride (EIPA), and methyl-β-cyclodextrin (MBCD). Different concentrations of each inhibitor were tested on DCs by incubating the cells with these inhibitors for 90 min. The LIVE/DEAD fixable dead cell stain kit (Thermo Fisher Scientific, MA, USA) was used according to the manufacturer’s instructions to evaluate the viability of DCs by flow cytometry. Cells without inhibitor treatments were used as controls.

### Effects of LSur on Dendritic Cell Migration

Groups of C57BL/6 mice (6–8 weeks of age) were injected with Sur or LSur (100 µg) in the left hind foot pad. Inguinal lymph nodes were dissected 6 and 24 h after injection. LNs were mechanically disaggregated to yield single cell suspensions. Cells were stained with FITC-conjugated anti-CD11c and PE-conjugated anti-CD40 antibodies and analyzed by flow cytometry.

### Immunization

Groups of C57BL/6 mice (6–8 weeks of age) were immunized with Sur or LSur (30 µg/dose) *via* footpad injection. Mice were injected with PBS (without antigens) and were used as controls. All animals were immunized two times at a 2-week interval with the same regimen.

### Enzyme-Linked Immunospot (ELISPOT) Assays

The mice were sacrificed 7 or 8 days after the boost vaccination and splenocytes were prepared. The frequency of IFN-γ-producing cells was determined using mouse IFN-γ ELISPOT kits (PB Pharmingen). All assays were performed according to the manufacturer’s procedures. In brief, 96-well plates with PVDF membranes (Millipore) were coated with capturing antibodies and then incubated at 4°C for 18 h. The plates were washed twice and blocked with RPMI medium supplemented with fetal bovine serum (10%) for 1 h to prevent non-specific binding in later steps. The splenocytes were seeded at a concentration of 5 × 10^5^ cells/well with a panel of 27 15-mer peptides with nine overlapping amino acids derived from human Sur (Table S1 in Supplementary Material). Triplicate wells were set up for each stimulation. Concanavalin A (5 µg/mL), control peptides, and media (no stimulation) were included as controls in parallel. After stimulation for 2 days at 37°C in a 5% CO_2_ humidified incubator, the cells were removed from the plates by washing three times with 0.05% (w/v) Tween 20 in PBS. Then, a biotinylated detection antibody (100 µL) was added to each well and the plates were incubated at 37°C for 2 h. The washing steps were repeated as above. After incubation at room temperature for 45 min with the avidin-horseradish peroxidase complex reagent, the plates were washed three times with 0.05% (w/v) Tween 20 in PBS and then three times with PBS alone. A 100-µL aliquot of 3-amine-9-ethylcarbazole (Sigma-Aldrich) staining solution was added to each well to develop the spots. The reaction was stopped after 1 h by placing the plates under tap water. The spots were counted using an ELISPOT reader (Cellular Technology Ltd., Shaker Heights, OH, USA).

### Animal Models

One week after the last immunization, the mice were inoculated with 2 × 10^5^ TC-1 cells in 0.2 mL of PBS in the left flank. Tumor growth was monitored by visual inspection and palpation. The tumor size was measured with a caliper, and the tumor volume was estimated by the formula *V* = width × length × (width + length)/2. The mice were sacrificed when the tumor volume reached 3000 mm^3^. For some experiments, the mice were intraperitoneally administered an isotype control antibody or an anti-CD8 antibody one day before tumor inoculation.

### Data Analysis

Statistical analysis was performed using GraphPad Prism software version 5.02 (GraphPad Software, San Diego, CA, USA), as described in each figure. Differences with *p* < 0.05 were considered to be statistically significant.

## Results

### Preparation and Characterization of Recombinant Lipidated Survivin

**Figure 1:**
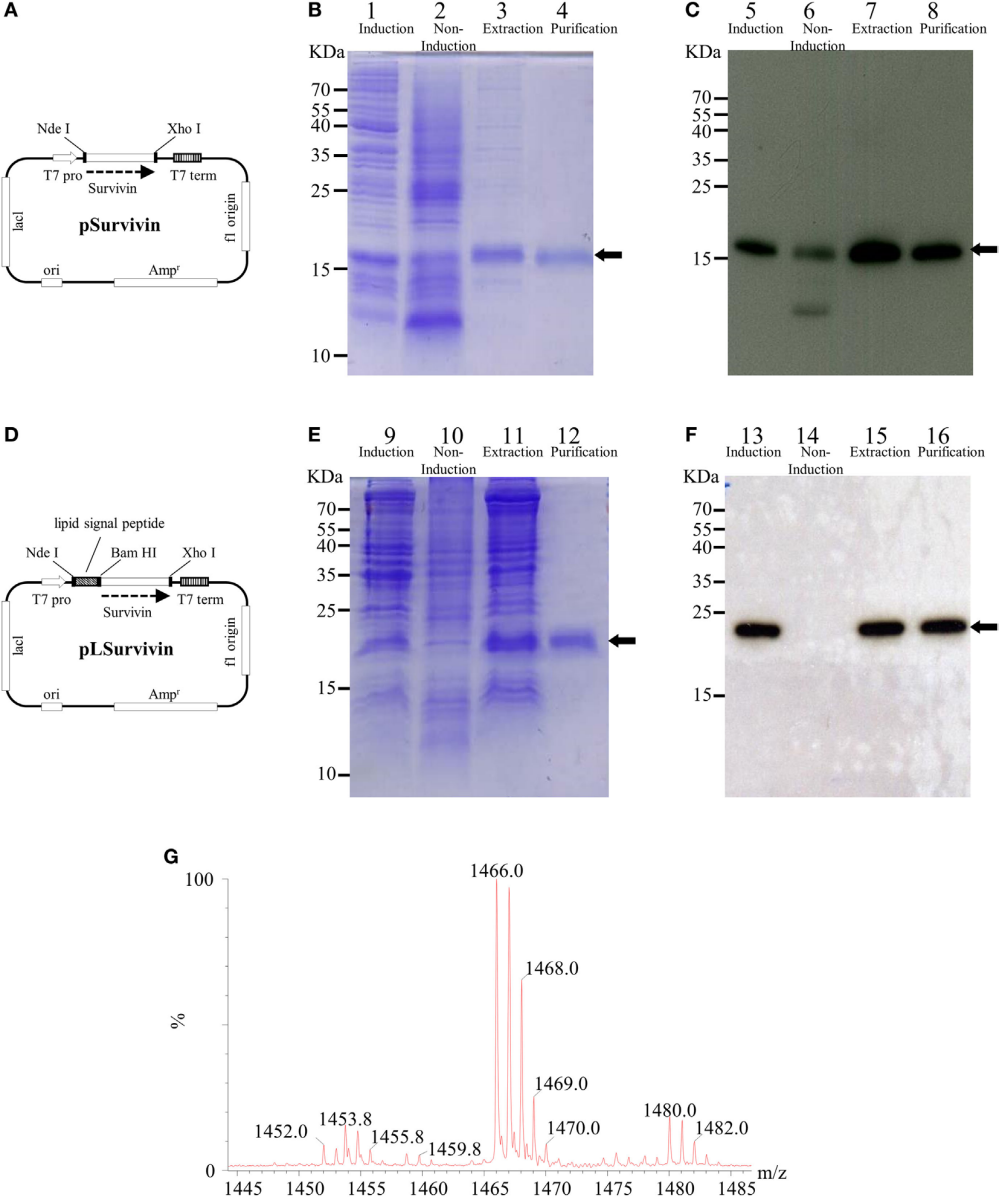
Production and purification of recombinant survivin and lipidated survivin. The DNA sequence encoding human survivin (AAC51660) was optimized for *Escherichia coli* codon usage and fully synthesized. The PCR product was cloned into a pER22b-based vector to generate the expression plasmid pSurvivin for the production of recombinant human survivin **(A)**. To construct pLSuvivin for the production of recombinant human lipidated survivin, pSurvivin was cloned into the pET22b-based vector with a lipid signal peptide in front of the survivin gene **(D)**. Both recombinant proteins contained an additional hexahistidine sequence (His-tag) at their C-termini and were expressed under the control of the T7 promoter. The purification of the survivin **(B,C)** and lipidated survivin protein **(E,F)** was monitored by 15% reducing SDS-PAGE followed by Coomassie Blue staining (lanes 1–4, 9–12) and immunoblotting with anti-His-tag antibodies (lanes 5–8, 13–16). The arrows indicate the electrophoretic positions of survivin or lipidated survivin in the gels or blots. Lanes 1, 5, 9, and 13: protein expression after isopropylthiogalactoside (IPTG) induction; lanes 2, 6, 10, and 14: protein expression in the absence of IPTG induction; lanes 3, 7, 11, and 15: extraction of protein from inclusion body; lanes 4, 8, 12, and 16: purified protein. **(G)** The *N*-terminal fragments of lipidated survivin were obtained and identified after trypsin digestion of lipidated survivin. The digested sample was analyzed on a Waters^®^ MALDI micro MX™ mass spectrometer. The MALDI-TOF MS spectra revealed the existence of three major peaks with *m/z* values of 1,452, 1,466, and 1,480.

The Sur gene was cloned into the pET-22b(+) expression vector (Novagen, Madison, WI, USA) using *Nde*I and *Xho*I sites to produce the pSurvivin plasmid for the production of recombinant human Sur. To produce LSur, the Sur gene was cloned into the pET-22b-based plasmid, which contained a lipid signal peptide, using *Bam*HI and *Xho*I sites to produce the pLSurvivin plasmid. As a result, both Sur and LSur contained an additional HHHHHH sequence (HsiTag) at the C-terminus and were expressed under the control of the T7 promoter (Figures 1A,D).

Survivin was purified using an IMAC column (Figure 1B, lanes 1–4) and detected with anti-His-Tag antibodies (Figure 1C, lanes 5–8). LSur was purified using a copper chelating sepharose column (Figure 1E, lanes 9–12) and detected with anti-His-Tag antibodies (Figure 1F, lanes 13–16). After removing the LPS, the residues of LPS in purified Sur and LSur were less than 60 EU/mg.

Next, we measured the exact mass of the *N*-terminal fragments of LSur. As shown in Figure 1G, three major peaks with *m*/*z* values of 1452.0, 1466.0, and 1480.0 were identified in the MALDI-TOF MS spectra. These peaks have been identified in other recombinant lipidated proteins and can be considered as lipidation signatures (33, 38–41). These results suggest that LSur contains a lipidated cysteine residue at its *N*-terminus.

### Functional Evaluation of Recombinant Lipidated Survivin

**Figure 2:**
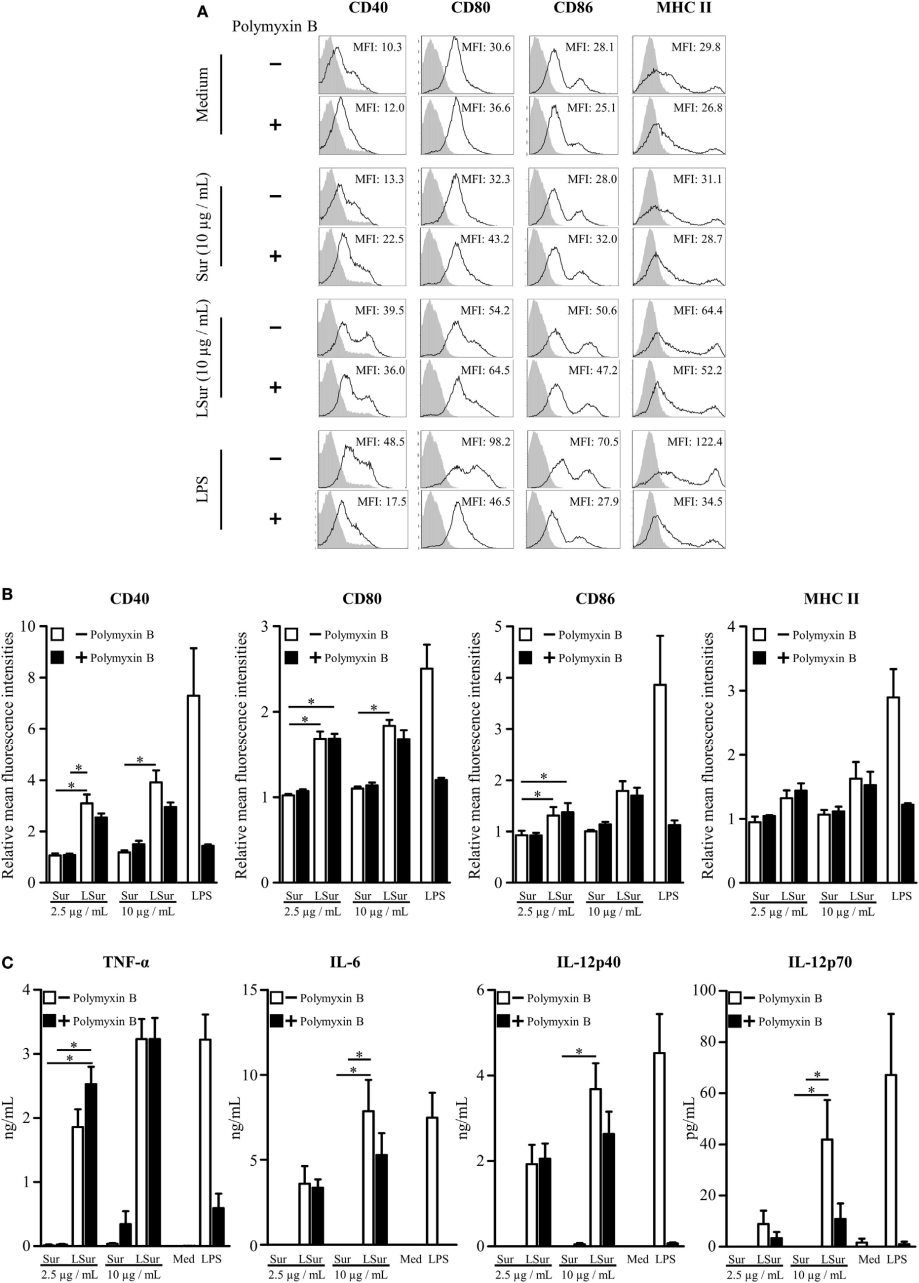
Activation of bone-marrow-derived dendritic cells induced by lipidated survivin. Mouse bone-marrow-derived dendritic cells were cultured in medium supplemented with survivin or lipidated survivin (2.5 or 10 µg/mL) in the presence or absence of polymyxin B (25 µg/mL). Dendritic cells stimulated with medium alone or lipopolysaccharide were used as controls. **(A)** After incubation for 20 h, the cells were harvested. The expression levels of CD40, CD80, CD86, and MHC II on a CD11c^+^ gated population were analyzed by flow cytometry. A representative experiment is shown. **(B)** The mean fluorescence intensity for cells stimulated with medium alone was defined as the basal expression level. The relative mean fluorescence intensities were plotted. The data represent the means ± SE of the mean from four independent experiments. **(C)** The supernatants were collected at 20 h after stimulation. The levels of TNF-α, IL-6, IL-12p40, and IL-12p70 were determined using ELISA kits. The data represent the means ± SE of the mean from four independent experiments. The statistical significance was determined using the Kruskal–Wallis test with Dunn’s multiple comparison test. *p < 0.05.

We (29) and others (24, 26–28) have demonstrated that bacterially produced lipoproteins can stimulate antigen-presenting cells *via* the toll-like receptors. BMDCs were stimulated with Sur or LSur to evaluate the functionality of LSur. The expression levels of CD40, CD80, CD86, and MHC II on BMDCs were analyzed by flow cytometry. As shown in Figure 2A, LSur upregulated the expression of CD40, CD80, CD86, and MHC II, while Sur (without lipidation) was ineffective at upregulating these molecules. To exclude the effect of residual endotoxin in recombinant protein preparation, polymyxin B was added to the recombinant proteins. Our data showed that there were no significant effects on Sur and LSur stimulation, but not LPS stimulation. The basal expression level was defined as the mean fluorescence intensity (MFI) of BMDCs stimulated with medium alone. The relative MFIs from four independent experiments are summarized in Figure 2B. These results suggest that upregulation of CD40, CD80, CD86, and MHC II expression was due to lipid moiety of LSur. In addition, LSur was able to stimulate the production of TNF-α, IL-6, IL-12p40, and IL-12p70 by BMDCs. Cytokine production was not abrogated in the presence of polymyxin B. In contrast, Sur (without lipidation) did not stimulate cytokine production (Figure 2C). These results indicate that LSur functions by activating BMDCs.

### Analysis of Uptake and Intracellular Trafficking of Recombinant Lipidated Survivin

**Figure 3:**
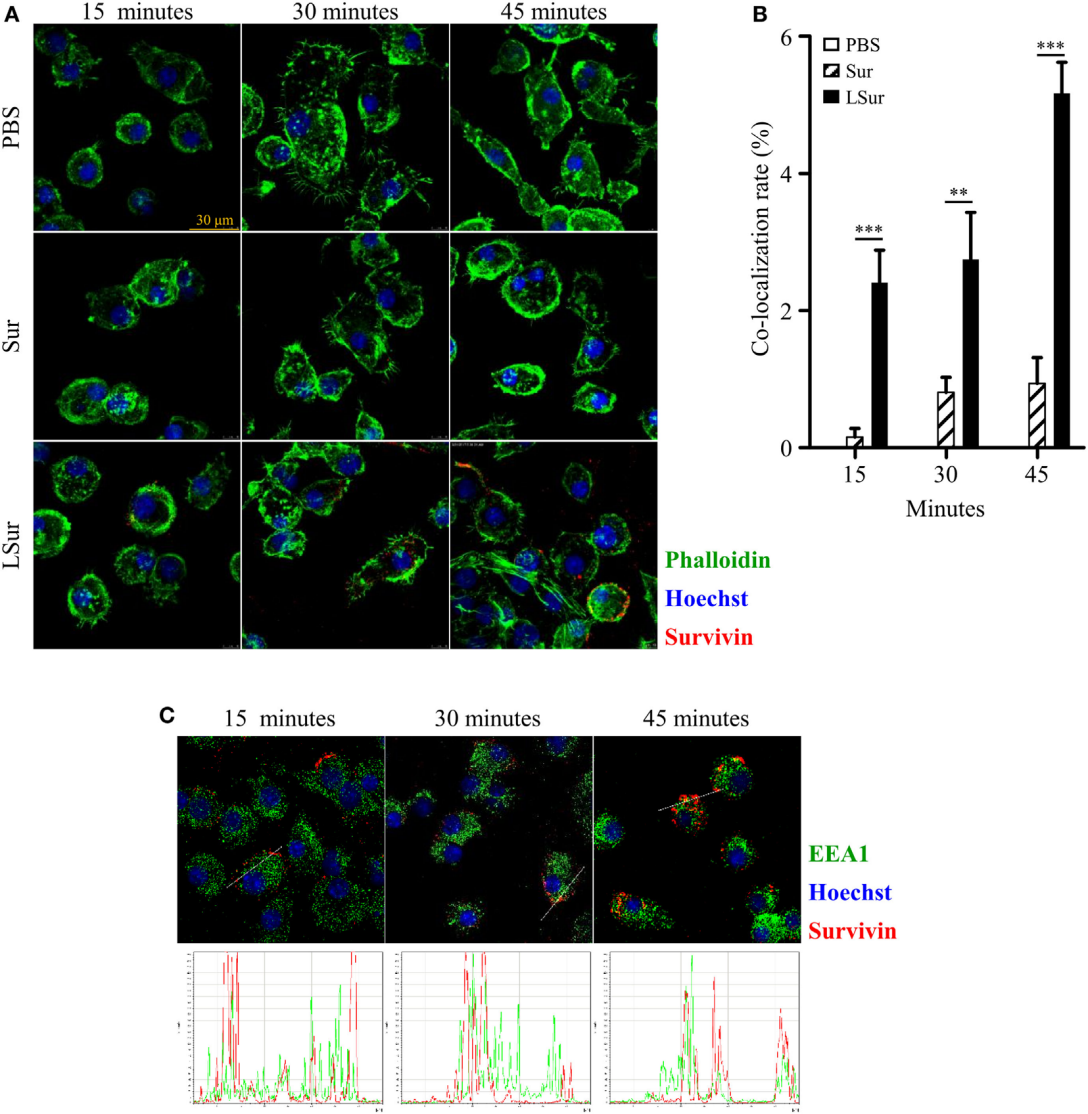
Recombinant lipidated survivin efficiently internalized by dendritic cells. Bone-marrow-derived dendritic cells were incubated with survivin or lipidated survivin (10 µg/mL) for the indicated time. Dendritic cells treated with PBS alone were used as controls. After incubation, the cells were washed, fixed, intracellularly stained with the indicated markers, and then analyzed by confocal microscopy. **(A)** The results are shown as phalloidin-labeled actin (green excitation), Hoechst staining for cell nuclei (blue excitation), and anti-survivin antibody staining (red excitation). **(B)** The co-localization of survivin and actin was quantified using the co-localization module of the Leica LAS AF software. The results are expressed as the percentages of co-localization (means ± SE; *n* = 8–10). The statistical significance was determined by Mann–Whitney test. **p < 0.01 and ***p < 0.001. **(C)** The results are shown as anti-EEA1 antibody staining (green excitation), Hoechst staining for cell nuclei (blue excitation), and anti-survivin antibody staining (red excitation).

In view of our finding on the activation of DCs by LSur (Figure 2), we next examined the uptake and intracellular localization of LSur. BMDCs were stimulated with LSur or Sur. Obviously, BMDCs took up a considerable amount of LSur and more efficiently than Sur (Figure 3A). The co-localization rates of Sur with actin were significantly higher in LSur-stimulated BMDCs than in Sur-stimulated BMDCs (Figure 3B). The cross-presentation of a soluble antigen depends on the location of the antigen in an early endosomal compartment (42). To investigate whether internalized LSur targeted the early endosomal compartment, we employed multiple staining analysis. Upon entry into the cells, LSur was rapidly delivered to organelles expressing the early endosomal marker EEA-1 in BMDCs only 15 min after stimulation. LSur colocalization with the early endosome was still detected 30 and 45 min after stimulation (Figure 3C). These results suggest that LSur was efficiently internalized by BMDCs and targeted the early endosomal compartment.

**Figure 4:**
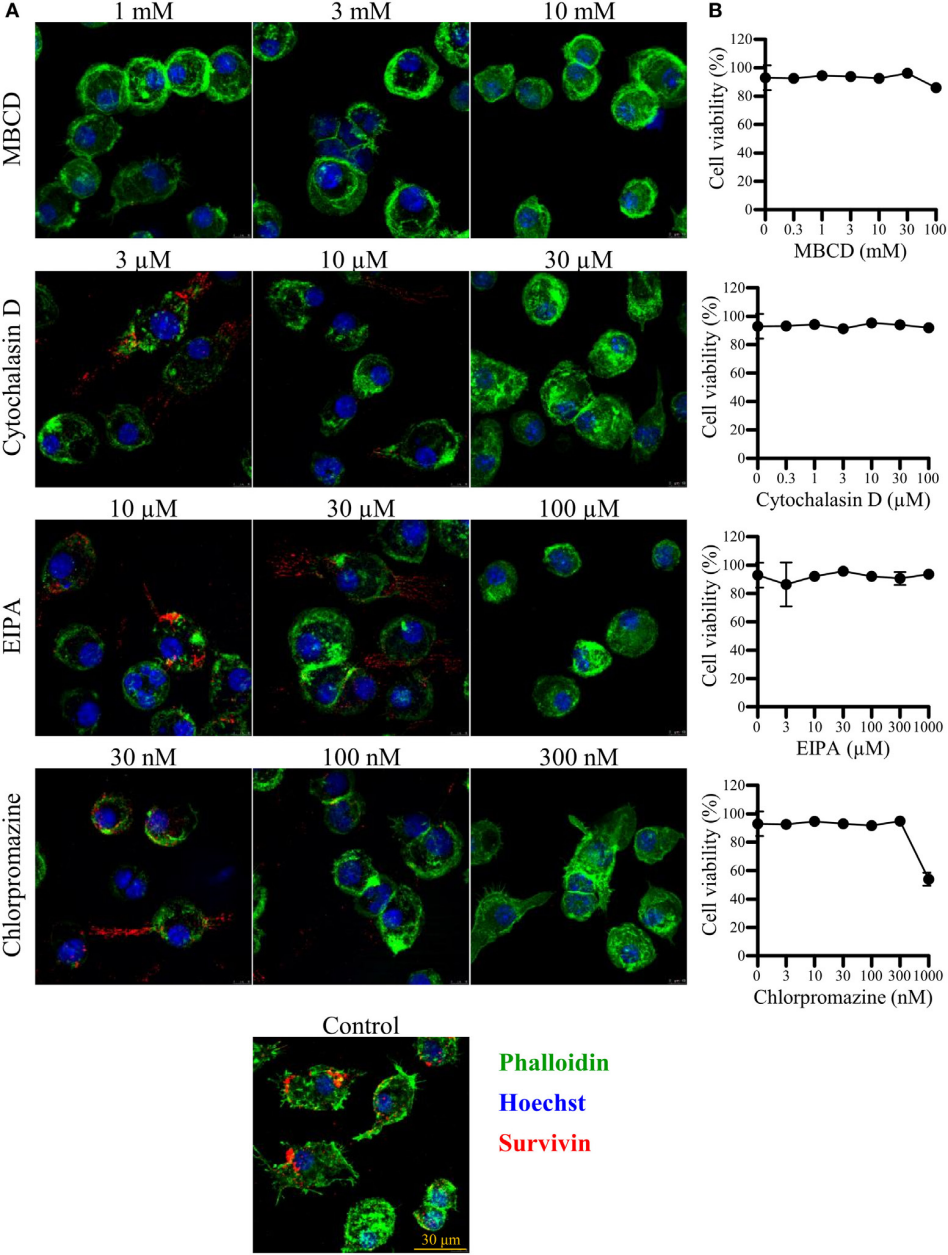
Lipidated survivin internalized by dendritic cells is reduced in the presence of endocytosis inhibitors. Bone-marrow-derived dendritic cells were incubated with lipidated survivin (10 µg/mL) for 45 min in the presence of the indicated endocytosis inhibitors. Dendritic cells treated without endocytosis inhibitors were used as controls. **(A)** After incubation, the cells were washed, fixed, intracellularly stained with the indicated markers, and then analyzed by confocal microscopy. The results are shown as phalloidin-labeled actin (green excitation), Hoechst staining for the cell nuclei (blue excitation), and anti-survivin antibody staining (red excitation). Data are representative of at least three images captured from two independent experiments. **(B)** Bone-marrow-derived-dendritic cells were incubated for 1.5 h with the indicated endocytosis inhibitors. Cell viability was assessed with the live/dead staining kit and evaluated by flow cytometry. Data are representative of two different experiments performed in duplicate.

To further examine the pathway of LSur uptake by BMDCs, we performed a detailed characterization of four commonly used endocytosis inhibitors (MBCD, cytochalasin D, EIPA, and chlorpromazine) on cell viability and endocytosis in BMDCs. LSur uptake by BMDCs was remarkably reduced following treatment with 1 to 10-mM MBCD. Moreover, the internalization of LSur by BMDCs was inhibited in the presence of cytochalasin D, EIPA, and chlorpromazine in a dose-dependent manner. No significant effects were observed at low concentrations (the left column of Figure 4A) of cytochalasin D (3 µM), EIPA (10 µM), and chlorpromazine (30 nM). LSur predominantly localized to the cellular boundaries at medium concentrations (the middle column of Figure 4A) of cytochalasin D (10 µM), EIPA (30 µM), and chlorpromazine (100 nM). Very little punctate staining was observed at high concentrations (the right column Figure 4A). To confirm that the reduction of internalized LSur was not mediated by cell death, the cell viabilities were assessed and were not significantly reduced within the concentration ranges used in the inhibitor treatments (Figure 4B). These results suggest that LSur is taken up by BMDCs *via* endocytosis.

### Elevation of Dendritic Cell Frequency in Draining Lymph Nodes by Injection With LSur

**Figure 5:**
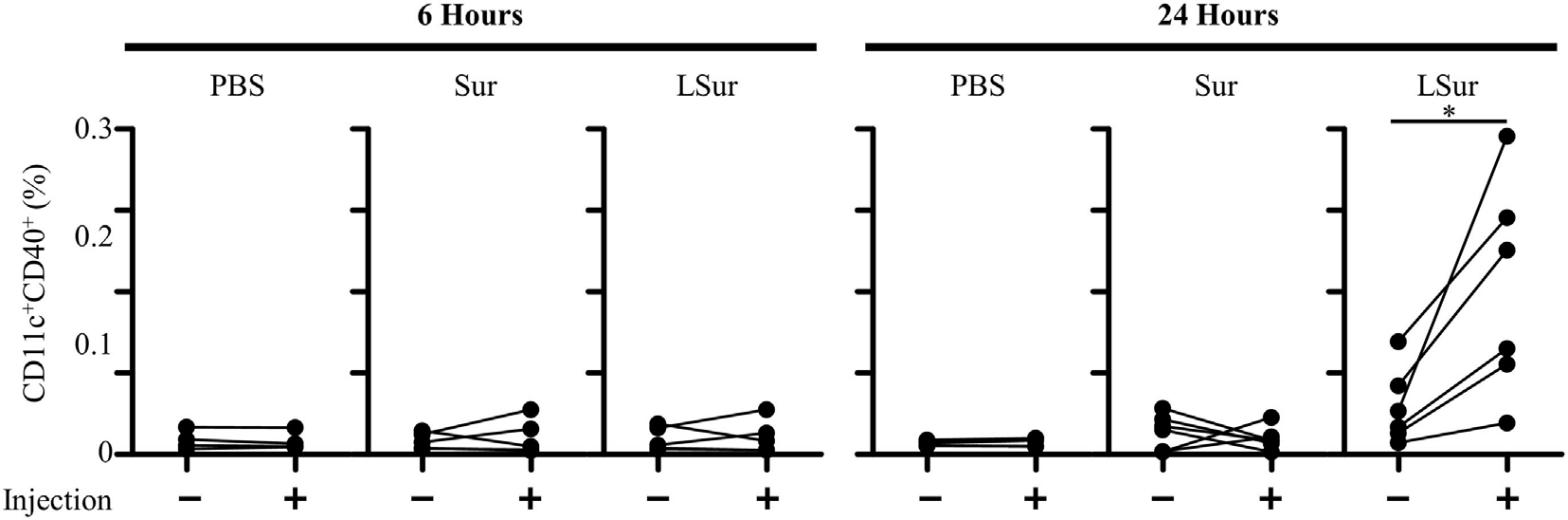
Recombinant lipidated survivin increases the frequency of dendritic cells in lymph nodes. Groups of C57BL/6 mice were injected with recombinant survivin or lipidated survivin (100 µg) in the left hind foot pad. Right (injection: −) and left (injection: +) inguinal lymph nodes were harvested 6 or 24 h after injection. The frequencies of CD11c^+^ CD40^+^ cells in the lymph nodes were analyzed by flow cytometry. The results were pooled from two independent experiments. The statistical significance was determined by a paired *t*-test. *p < 0.05.

Dendritic cells capture antigens in the peripheral tissues and then migrate to secondary lymphoid organs. Lymph nodes are the critical sites where DCs communicate with lymphocytes to coordinate adaptive immune responses. To investigate whether LSur could increase the frequencies of DC in lymph nodes, mice were injected LSur, Sur, or PBS in their left foot pads. The frequencies of CD11c^+^ CD40^+^ cells in both the left (the injected site) and right (the non-injected site) inguinal lymph nodes were analyzed by flow cytometry at 6 or 24 h after injection. The injection of LSur increased the frequencies of CD11c^+^ CD40^+^ cells in the left inguinal lymph nodes in comparison to the right inguinal lymph node in the same mouse at 24 h, but not 6 h, after injection. We further examined the phenotype of these increased CD11c^+^ CD40^+^ cells and found that the majority of CD11c^+^ CD40^+^ cells were also MHC II^+^ CD86^+^ cells (Figure S1 in Supplementary Material). The frequencies of CD11c^+^ CD40^+^ cells in lymph nodes were not altered after 24 h in mice injected with Sur; these cell frequencies were also similar to those of mice injected with PBS (Figure 5). These results indicate that the injection of LSur can increase CD11c^+^ CD40^+^ cells in the draining lymph node.

### Induction of Antitumor Immunity by Immunization With LSur Without Exogenous Adjuvant Formulation

**Figure 6:**
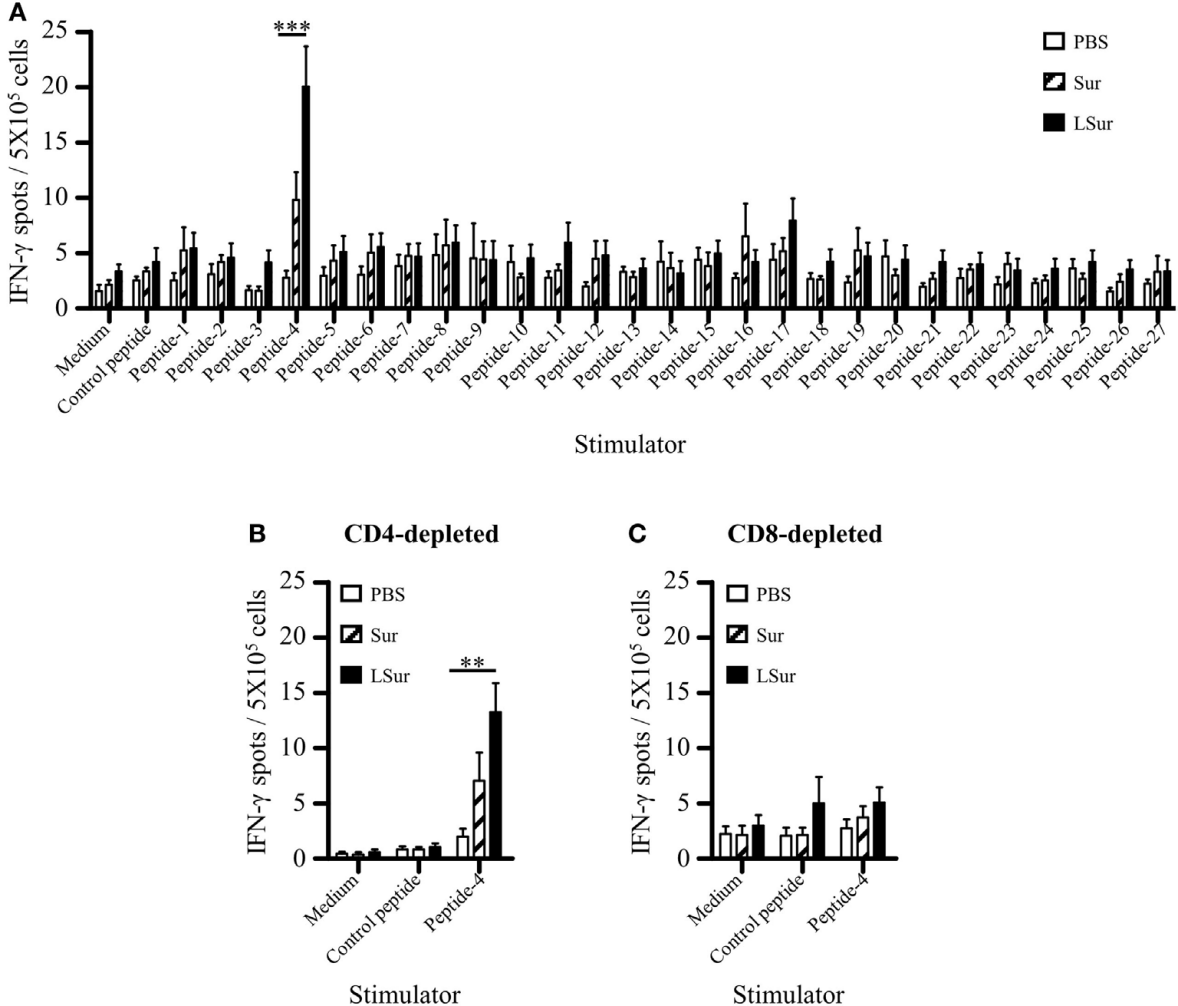
Recombinant lipidated survivin induces CD8^+^ T-cell responses. Groups of C57BL/6 mice (*n* = 4/group) were immunized twice with 30 µg of recombinant survivin or lipidated survivin at a 2-week interval. Mice immunized with PBS were used as controls. **(A)** Splenocytes were prepared 1 week after the last immunization. A panel of 15-mer overlapping peptides that covered the entire sequence of survivin was used to stimulate splenocytes. **(B)** CD4-depleted or **(C)** CD8-depleted splenocytes were stimulated with peptide-4. The frequencies of IFN-γ-producing cells in spleens were determined using mouse IFN-γ Enzyme-Linked Immunospot kits. Data represent the means ± SE of the mean. The results are one of two representative experiments. The statistical significance was determined using the Kruskal–Wallis test with Dunn’s multiple comparison test. **p < 0.01 and ***p < 0.001.

Groups of C57BL/6 mice were immunized with PBS, Sur, or LSur two times at a 2-week interval. One week after the last immunization, splenocytes from immunized mice were assayed by ELISPOT to evaluate T-cell responses against Sur. A panel of 27 overlapping peptides for Sur was used to stimulate IFN-γ production. Splenocytes from mice immunized with PBS induced a background level of IFN-γ secretion for all the peptides stimulations. A dominant peptide (peptide 4) was found to induce IFN-γ production in mice immunized with LSur (Figure 6A). To further test whether peptide 4 was CD4^+^ T-cell or CD8^+^ T-cell dependent in mice immunized with LSur, CD4-depleted and CD8-depleted splenocytes were used to assay IFN-γ production after stimulation by peptide 4. The specific IFN-γ responses were still present in CD4-depleted cells after stimulation with peptide 4 (Figure 6B). In contrast, the specific IFN-γ responses disappeared in CD8-depleted cells after stimulation with peptide 4 (Figure 6C). These results suggest that LSur is capable of triggering CD8^+^ T-cell responses in mice.

**Figure 7:**
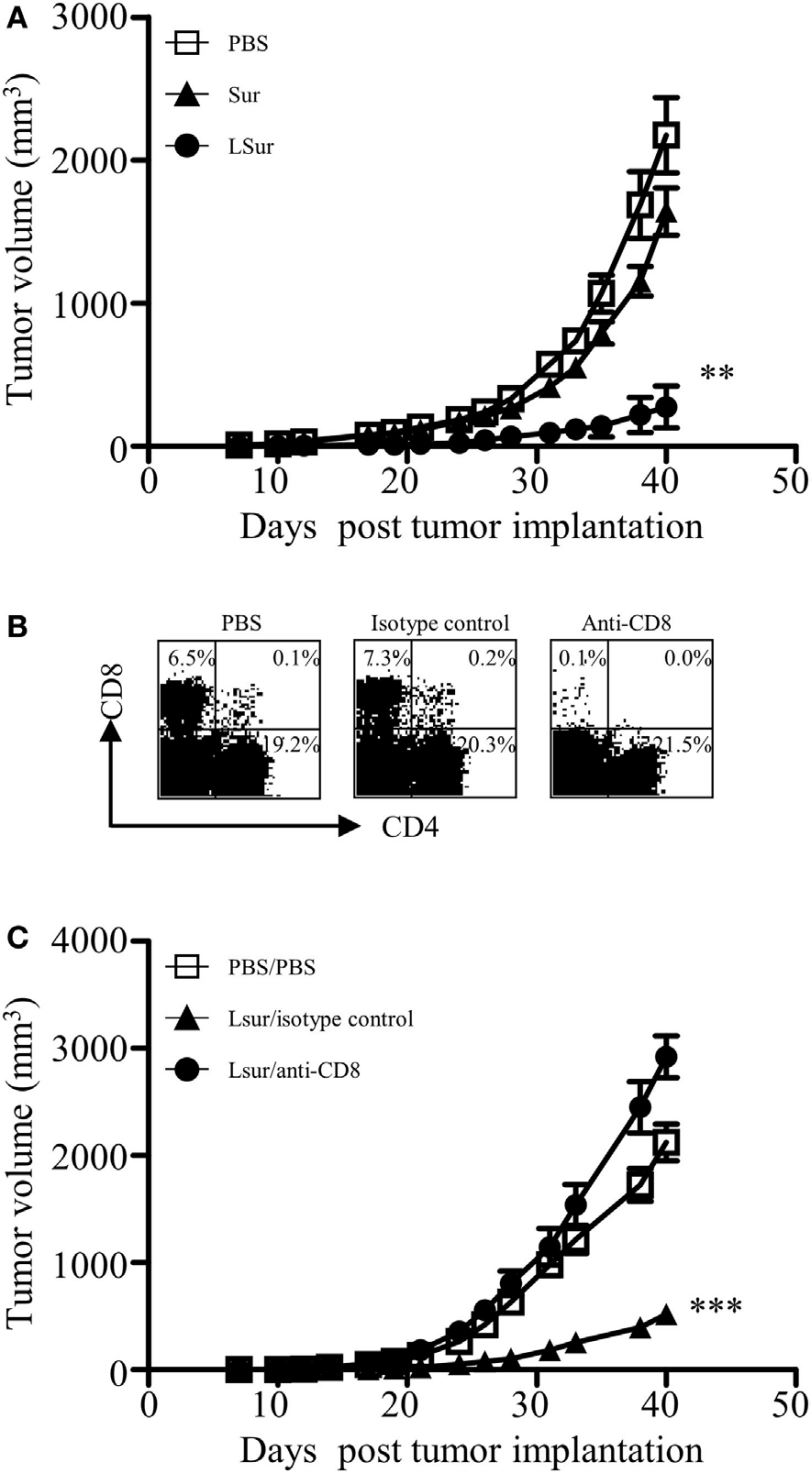
CD8^+^ T cells play a major role in antitumor response induced by recombinant lipidated survivin. Groups of C57BL/6 mice were immunized twice with 30 µg of recombinant survivin or lipidated survivin at a 2-week interval. Mice immunized with PBS were used as controls. One week after the last immunization, mice were inoculated with 2 × 10^5^ TC-1 cells. **(A)** Tumor growth was monitored (*n* = 5/group). **(B)** One day before tumor inoculation, mice were administered intraperitoneally with an isotype control antibody or an anti-CD8 antibody. Mice immunized with PBS and depleted with PBS were used as controls. Depletion of CD8^+^ T cells was confirmed by flow cytometry of splenocytes from one mouse in each group. **(C)** Tumor growth after depletion was monitored (*n* = 6/group). The results are one of two representative experiments. Statistics were calculated using a Kruskal–Wallis test with Dunn’s multiple comparison test on the tumor volume at 40 days post tumor implantation. **p < 0.01 compared with PBS and ***p < 0.001 compared with isotype control.

Given the induction of CD8^+^ T-cell responses elicited by vaccination with LSur, we evaluated the vaccinated mice for an *in vivo* antitumor effect against challenge by TC-1 cells. One week after the last vaccination, animals were challenged with TC-1 cells. As shown in Figure 7A, mice immunized with Sur were not protected, and the observed tumor progression was similar to that of the PBS-vaccinated animals. In contrast, mice immunized with LSur displayed significantly inhibited tumor growth (*p* < 0.01) compared with the PBS-vaccinated group. These results demonstrate that immunization with LSur is able to induce antitumor responses and inhibit tumor growth.

To further determine whether CD8^+^ T cells were responsible for the *in vivo* antitumor effect, CD8^+^ T cells were depleted using anti-CD8 antibodies one day before tumor inoculation. Depletion of CD8^+^ T lymphocyte subsets from mice *in vivo* was confirmed by flow cytometry 24 h after treatment with anti-CD8 antibodies (Figure 7B). The antitumor effect in LSur-vaccinated mice was abolished in anti-CD8 antibody-depleted mice but not in isotype antibody-depleted mice (Figure 7C). These results suggest that the CD8 population is mainly responsible for mediating *in vivo* antitumor responses in LSur-vaccinated mice.

## Discussion

The induction of cytotoxic T-cell responses is a crucial prerequisite for cancer immunotherapy *via* the use of intracellular antigens. The results presented here delineate the feasibility of using LSur to induce antitumor immunity. Moreover, the results illuminate the application of recombinant lipidated antigens in vaccine development.

The activation of DCs is considered a critical step in the induction of immune responses. Upon activation, DCs can present processed peptides with MHC and costimulatory molecules on their surface. According to the two-signal model, the interaction of TCR with peptide-MHC complexes confers specificity to T-cell activation and synergizes with costimulatory signaling but also results in T-cell unresponsiveness without costimulatory signaling (43). DCs employ receptors of the innate immune system, such as toll-like receptors, to sense microbial components during pathogen invasion (44, 45). These pathogen-derived products provide danger signals to activate DCs, which then enhance costimulatory molecule expression and cytokine production (33,  38, 40, 41, 46, 47). We showed that LSur, but not Sur, induced the activation of BMDCs, as evidenced by the secretion of IL-6, TNF-α, and IL-12 (Figure 2A) and the upregulated expression of CD40, CD80, CD86, and MHC II (Figure 2B). The activation of DCs by LSur favors the induction of Sur-specific T-cell responses.

An important procedure in the induction of adaptive immunity is the internalization of antigens by antigen-presenting cells. We showed that compared with Sur, LSur was internalized considerably more by BMDCs (Figures 3A,B). However, the mechanisms that mediate LSur uptake are unclear. In this study, we showed that Cytochalasin D, which affects most endocytic pathways (48), reduced LSur internalization by BMDCs in a dose-dependent manner (Figure 4). These results suggest that endocytosis serves an important pathway for LSur internalization. Endocytosis can be mediated by distinct routes (49). The most extensively described routes are lipid raft/caveolae-mediated endocytosis, clathrin-coated pits, and macropinocytosis/phagocytosis, three routes that are sensitive to MBCD, chlorpromazine, and EIPA inhibitors, respectively (48). LSur internalization was suppressed in the presence of these endocytosis inhibitors (Figure 4). These results suggest that LSur endocytosis by BMDCs can be mediated by multiple routes.

Dendritic cells, which are recognized as professional antigen-presenting cells, take up antigens in peripheral tissues and carry antigens to the draining lymph nodes (20–23). After entering the draining lymph nodes, these antigen-bearing DCs not only present the antigens to T cells but also deliver the antigens to lymph node-resident DCs. Lymph node-resident DCs receive antigens from migratory DCs and then efficiently present the antigens to T cells (50). We show that 24 h after injection, the number of DCs increased in the draining lymph node of LSur injected sites compared with the uninjected sites in the same mice. In addition, the increased DCs expressed not only CD11c and CD40 but also MHC II and CD86 (Figure S1 in Supplementary Material). In contrast, there was no significant change in the draining lymph node of sites injected with Sur or PBS (Figure 5). The induction of immunity or tolerance is determined by the presence or absence of danger signals when antigens are captured by professional antigen-presenting cells (51). We confirmed that LSur is able to enhance the production of inflammatory cytokines and the expression of costimulatory molecules by DCs (Figure 2). Moreover, compared with Sur, LSur was efficiently internalized by BMDCs (Figure 3A). As discussed above, these results demonstrate that LSur more efficiently activates T cells than its non-lipidated counterpart.

The cross-presentation of exogenous antigens is mainly carried out by DCs (52, 53) and plays an important role in antitumor immune responses. Exogenous antigens are taken up by DCs through endocytic and phagocytic pathways. The intracellular destination of an internalized antigen is a critical factor to determine the presentation efficiency. It has been shown that the transport of exogenous antigens to the early endosomal compartment results in more efficient cross-presentation (42, 54–56). After uptake by DCs, LSur was routed to the early endosome, as indicated by its co-localization with the early endosomal marker EEA-1 (Figure 3C). In line with this result, we showed that LSur stimulated CD8^+^ T-cell responses in the absence of an exogenous adjuvant (Figure 6).

In this study, we use human Sur as the model antigen. Human Sur is very similar to murine Sur. The identity of amino-acid sequence between human Sur and murine Sur is greater than 83% (Figure S2 in Supplementary Material). We took this advantage and used the TC-1 mouse tumor model. Notably, antitumor responses were elicited in mice after immunization with LSur (Figure 7A). We also demonstrated that CD8 cells mediate *in vivo* antitumor responses (Figure 7C). It is likely that LSur induces cross reactivity to murine Sur.

In summary, our study shows that LSur is efficiently internalized by DCs and delivered to early endosome for cross-presentation, thus enhancing CD8^+^ T-cell responses. Our findings indicate that LSur can be exploited to stimulate protective antitumor immunity, suggesting that lipidated tumor antigens may be a promising approach for inducing cytotoxic T lymphocyte responses to inhibit tumor growth.

## Ethics Statement

This study was carried out in accordance with the recommendations of Taiwan’s Animal Protection Act. The protocol was approved by the Animal Committee of the National Health Research Institutes.

## Author Contributions

S-JL, C-HL, and H-WC contributed to the conception and design of the experiments. C-YC, Y-JC, and C-CW performed the experiments in the manuscript. All authors contributed to the analysis and interpretation of the results. S-JL, C-HL, and H-WC led the manuscript writing. All authors participated to manuscript writing, editing, and critical reviewing.

## Conflict of Interest Statement

S-JL, C-HL, and H-WC are named on patents relating to the lipidated survivin for prevention and treatment of cancers. All other authors declare that the research was conducted in the absence of any commercial or financial relationships that could be construed as a potential conflict of interest.
