# Supplementary material for: Efficient Uptake of Recombinant Lipidated Survivin by Antigen-Presenting Cells Initiates Antigen Cross-Presentation and Antitumor Immunity
Source: Front Immunol. 2018 Apr 23;9:822. doi: 10.3389/fimmu.2018.00822 (PMC5932405; doi:10.3389/fimmu.2018.00822)
Supplement: Supplementary file 1 [file data_sheet_1.docx]

***Supplementary Material***

**Efficient uptake of recombinant lipidated survivin by antigen presenting cells initiates antigen cross-presentation and antitumor immunity**

**Chen-Yi Chiang, Yi-Jyun Chen, Chiao-Chieh Wu, Shih-Jen Liu, Chih-Hsiang Leng, Hsin-Wei Chen^*^**

*** Correspondence:** Hsin-Wei Chen ([chenhw@nhri.org.tw](mailto:chenhw@nhri.org.tw))

Supplementary Table 1. Peptides used for T cell stimulation.

| **Name** | **Sequence** |
| --- | --- |
| Peptide-1 | MGAPTLPPAWQPFLK |
| Peptide-2 | LPPAWQPFLKDHRIS |
| Peptide-3 | PFLKDHRISTFKNWP |
| Peptide-4 | HRISTFKNWPFLEG |
| Peptide-5 | FKNWPFLEGCACTPE |
| Peptide-6 | FLEGCACTPERMAEA |
| Peptide-7 | ACTPERMAEAGFIH |
| Peptide-8 | RMAEAGFIHCPTENE |
| Peptide-9 | GFIHCPTENEPDLAQ |
| Peptide-10 | PTENEPDLAQCFFCF |
| Peptide-11 | PDLAQCFFCFKELEG |
| Peptide-12 | CFFCFKELEGWEPDD |
| Peptide-13 | KELEGWEPDDDPIEE |
| Peptide-14 | WEPDDDPIEEHKKHS |
| Peptide-15 | DPIEEHKKHSSGCAF |
| Peptide-16 | HKKHSSGCAFLSVKK |
| Peptide-17 | SGCAFLSVKKQFEEL |
| Peptide-18 | LSVKKQFEELTLGEF |
| Peptide-19 | FEELTLGEFLKLDR |
| Peptide-20 | TLGEFLKLDRERAKN |
| Peptide-21 | LKLDRERAKNKIAKE |
| Peptide-22 | ERAKNKIAKETNNKK |
| Peptide-23 | KIAKETNNKKKEFEE |
| Peptide-24 | TNNKKKEFEETAKKV |
| Peptide-25 | KEFEETAKKVRRAIE |
| Peptide-26 | TAKKVRRAIEQLAAM |
| Peptide-27 | RRAIEQLAAMDLE |


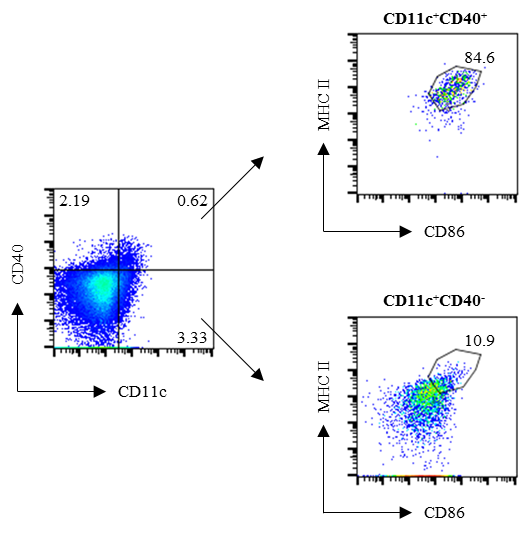


**Supplementary Figure 1. Expression profiles of MHC II and CD86 in the CD11c^+^CD40^+^ and CD11c^+^CD40^-^ subsets.** C57BL/6 mice were injected with recombinant lipidated human survivin (100 μg) in the left hind foot pad. The inguinal lymph node from the injection site was harvested 24 hours after injection. MHC II and CD86 expression profiles in the CD11c^+^CD40^+^ and CD11c^+^CD40^-^ subsets were analyzed by flow cytometry. Results are representative of 3 mice.

Human survivin 1 50

Human survivin MGAPTLPPAWQPFLKDHRISTFKNWPFLEGCACTPERMAEAGFIHCPTEN

Murine survivin MGAPALPQIWQLYLKNYRIATFKNWPFLEDCACTPERMAEAGFIHCPTEN

Consensus MGAP LP WQ FLK HRIATFKNWPFLE CACTPERMAEAGFIHCPTEN

Human survivin 51 100

Human survivin EPDLAQCFFCFKELEGWEPDDDPIEEHKKHSSGCAFLSVKKQFEELTLGE

Murine survivin EPDLAQCFFCFKELEGWEPDDNPIEEHRKHSPGCAFLTVKKQMEELTVSE

Consensus EPDLAQCFFCFKELEGWEPDD PIEEHKKHS GCAFLSVKKQ EELTL E

Human survivin 101 142

Human survivin FLKLDRERAKNKIAKETNNKKKEFEETAKKVRRAIEQLAAMD

Murine survivin FLKLDRQRAKNKIAKETNNKQKEFEETAKTTRQSIEQLAA--

Consensus FLKLDR RAKNKIAKETNNK KEFEETAK R AIEQLAA

**Supplementary Figure 2. Sequence alignment of human (AAC51660) and murine (AAD26199) survivin using Vector NTI.**
